# Arctic passages: liminality, Iñupiat Eskimo mothers and NW Alaska communities in transition

**DOI:** 10.3402/ijch.v72i0.21199

**Published:** 2013-08-05

**Authors:** Lisa Llewellyn Schwarzburg

**Affiliations:** UAF Interdisciplinary Program Cross-Cultural and Indigenous Studies, Rural and Indigenous Health Policy, University of Alaska Fairbanks, Fairbanks, AK, USA

**Keywords:** Alaska Native birth, embodiment, indigenous birth, Iñupiat values, maternal identity work, maternal transport policy, women-centred ethnography

## Abstract

**Background:**

While the primary goal of the NW Alaska Native maternal transport is safe deliveries for mothers from remote villages, little has been done to question the impact of transport on the mothers and communities involved. This study explores how presence of Iñupiat values influences the desire of indigenous women of differing eras and NW Alaska villages to participate in biomedical birth, largely made available by a tribal health-sponsored transport system.

**Objective:**

This paper portrays how important it is (and why) for Alaska Native families and women of different generations from various areas of Iñupiat villages of NW Alaska to get to the hospital to give birth. This research asks: How does a community's presence of Iñupiat values influence women of different eras and locations to participate in a more biomedical mode of birth?

**Design:**

Theoretical frameworks of medical anthropology and maternal identity work are used to track the differences in regard to the maternal transport operation for Iñupiat mothers of the area. Presence of Iñupiat values in each of the communities is compared by birth era and location for each village. Content analysis is conducted to determine common themes in an inductive, recursive fashion.

**Results:**

A connection is shown between a community's manifestation of Iñupiat cultural expression and mothers’ acceptance of maternal transport in this study. For this group of Iñupiat Eskimo mothers, there is interplay between community expression of Iñupiat values and desire and lengths gone to by women of different eras and locations.

**Conclusions:**

The more openly manifested the Iñupiat values of the community, the more likely alternative birthing practices sought, lessening the reliance on the existing transport policy. Conversely, the more openly western values are manifested in the village of origin, the less likely alternative measures are sought. For this study group, mothers from study villages with openly manifested western values are more likely to easily acquiesce to policy, and “make the best” of their prenatal travel.

Just as the Northwest Passage, a sea route through the Arctic Ocean, is fraught with physical, political and geographical obstacles, so are the social passages of peoples inhabiting the Arctic region of Northwest Alaska.

This study – seated in the combined theoretical frameworks of Medical Anthropology (using the concepts of liminality, communitas and embodiment) and concepts of maternal and Alaska Native identity – explores the impact of the presence of Iñupiat values of a community on the expressed desired access to maternal care among the study population. The aim of this paper is to highlight major findings from a larger dissertation study (1) and discuss implications for the policy.

It also responds to the call for *women-centred ethnographic study* of birth by the World Health Organization (2):

Our results highlight the need to thoroughly explore and address context-specific causes of variable use of maternal health care if safe motherhood is to become a reality … . (p. 816–7)

and Centers for Disease Control (3):

Further evaluations of these projects [health improvement initiatives], especially in AN communities, could provide further evidence to understand the underlying causes of the persistent disparity between AN and non-AN postneonatal mortality rates. (p. 2)

These agencies point to structural determinants of health like socio-political systems and unequal distribution of resources as underlying causes of poor child health (4). In their review of multiple vertical maternal and child health programs, McCoy et al. (4) cautioned against exclusive use of evidence-based data at the expense of losing meaningful, important context, specifically regarding maternal mortality:

… While the direct causes of death are classified biomedically … and prioritise the availability of emergency obstetric services, underpinning these deaths may be lack of access to health care due to economic or social discrimination or marginalization, which requires more of a broader public health approach. (91)

To address a lack of qualitative information concerning the maternal care services as utilized by the NW Alaska Native Iñupiat population, research questions addressed in this article include:

- How do the experiences of different generations of mothers, transport situations and communities compare (particularly in regard to expression of Iñupiat values and social embodiment, *liminality* and associated *communitas*)?
- How do expectant women, families and community members perceive the Alaska Native Village Maternal Transport (ANVMT) policy today? What are their main concerns?

This study shows how Alaska Native families and women of different generations from various areas of mostly-Iñupiat^1^ villages of NW Alaska consider the importance of getting to the urban hospital to birth. Information concerning usage of health care derived directly from the viewpoint and voices of these participants helps inform agencies tracking health disparities in this area. Many women desiring change are calling for greater communication with the people in charge of making their health care decisions. As one Point Hope mother of three describes: “… I think it would be better for us [Maniilaq-area Iñupiat mothers] to talk with the people [caregivers in charge of making decisions] ourselves, instead of having the community health aide do it for us” (5).

## Definitions

### ANVMT policy

A system of air travel that enables Alaska Native expectant mothers from remote villages to access maternal health care by flying these women out (about 4 weeks prior to delivery) to give birth at a regional hub or urban Alaska Native hospital, and return to their villages with their babies.

The application of this policy varies from region-to-region, depending on the level of care available in the region's hub at time of expected delivery; the determination and perception of risk involved for the mothers and babies; and type of health care coverage being used (private or Medicaid-based insurance, or tribal consortium coverage).

This policy also provides for maternal stay in either a dorm-like facility at the urban hospital or for stay in a nearby hotel. Depending on the situation (age, severity of condition), escort travel and lodging can possibly be covered under this system, as well. An indirect impact of this arrangement is the access to urban shopping and visits with Anchorage relatives facilitated by the somewhat lengthy stay. Some mothers can also regard travel and stay as an undue burden – citing other parenting responsibilities, work or relationship conflicts, or being away from home for so long (1).

### Liminality

An anthropological concept concerning a state of “in-betweeness,” introduced by van Gennep (6) in descriptions of *Rites of Passage* and further developed by Turner (7) in the late 1960s. He describes the phases through which the *liminoid* (individual undergoing change) proceeds, going from a state of structure (guided by cultural norms), to a state of anti-structure (where normal guidelines don't apply), and finally, back into the structured social environment as a transformed member of the group. Pregnancy is often viewed as a rite of passage and a state of liminality involving social movement of a community member from status of non-mother to mother (8), with changes in roles and expectations in that process.

Many authors use the related concept of *communitas* to describe the camaraderie and closeness that might be felt among fellow liminoids that might effectively move them toward structure and re-entry into their communities. Trosset (9) counts Welsh ethnic affinity as communitas, adding that it can also describe communities having experienced common life experiences that “generate similar habitual dispositions.” This study's findings include a sense of communitas at play in study villages openly expressing Iñupiat values. The concepts of liminality and communitas are used in this research in reference to both the individuals experiencing the transitions into motherhood, and the communities as they experienced change from Iñupiat and western cultural influences. Guar and Patnaik (10), in their work among the indigenous Korwa community in Central India, found health-generating and health-threatening attributes of life for the Korwa informants moving from hill forests to lowland villages. Similar changes are described by Iñupiat women of MSA birthing in earlier eras in comparison to their counterparts’ birth accounts from today. Also, while Douglas (11) does not specifically employ the concept of liminality, she found creation of what she termed “non-modern” traditionalism (in a sense, communitas), in the treatment of birth among the Inuit populations in the First Nations Canadian village of Nunavik – that arguably serves as another example of a liminal process at the community level.

### Identity-work

A process of techniques that individuals use to discursively construct a social image of self. This process, while not necessarily so, can also take place during a liminal phase, as explained in Gimlin's (12) study of cosmetic surgery patients. The current study concentrates on the intersection of maternal identity work (13,14) with Iñupiat maternal identity construction – and community identity work as a specific Iñupiaq village. Individual and community-level concepts of identities are featured in this study's treatment of indigeneity.

Some authors have found ties between level of traditionalism and disease risk and protective behaviors among indigenous populations (15,16). This study looks at how women and their respective communities assess their risks. Similar to Erikson's (17) treatment of the importance of life transitions among former East Germans birthing in reunified Germany, risk assessment during prenatal care can actually be a form of social embodiment.

### Embodiment

A conceptualization of the body as a reflection of the conditions of its existence, often superseding what individuals may not be able, allowed, or wants to express. Epidemiologists using this construct understand the body as a reflection of the body politic (18).

In her thesis on the routinization of prenatal testing in Ottawa, Ontario, Shoemaker (19) uses Foucault's (20) premise that women are led to participate in the medicalization of pregnancy through normalization of government surveillance of health. As in the process described by Erikson (17), Alaska Native mothers in this study have expressed feelings of being labelled troublesome when not following the processes deemed necessary in their biomedical, geopolitical environment.

Therefore, embodiment as Alaska Native pregnant women from the Arctic bears meaning for them both internally, carrying babies of the Iñupiat Eskimo community to which they belong, and externally from the social milieus of their villages and outside state, tribal, and national bureaucracies that provide their care.

This look into Iñupiat mothers’ considerations of the ANVMT policy as it operates in their home village, alongside current health statistics for the area reveals a backdrop of an Arctic brand of embodiment. Undercurrents of power struggle, inequality and discrimination appear if appointments are missed or relationship issues cause problems. Even well-meaning advice can be construed as an effort to shame or embarrass a patient.

### Presence of Iñupiat values

Features of Iñupiat values present in each community were noted for this research in similar manner used by Coe et al. (15) in their work on traditionalism [their term] as a factor in disease risk and protective behaviors among Hopi women living on the reservation.

Using 3 dimensions of native culture: language usage; cultural participation; and village involvement with Alaska Native-based activities, features were described for each study village. Observations of signs in public locations, local speech in Iñupiaq and uniquely-Iñupiat commemoration of religious and national holidays were included in this assessment. Specific cultural participation refers to celebrations, life events, artefacts and dances that centre on Iñupiat subsistence lifestyle. The “village involvement” section describes the manifestations of Iñupiat culture present in everyday life.

## Methods

**Fig. 1:**
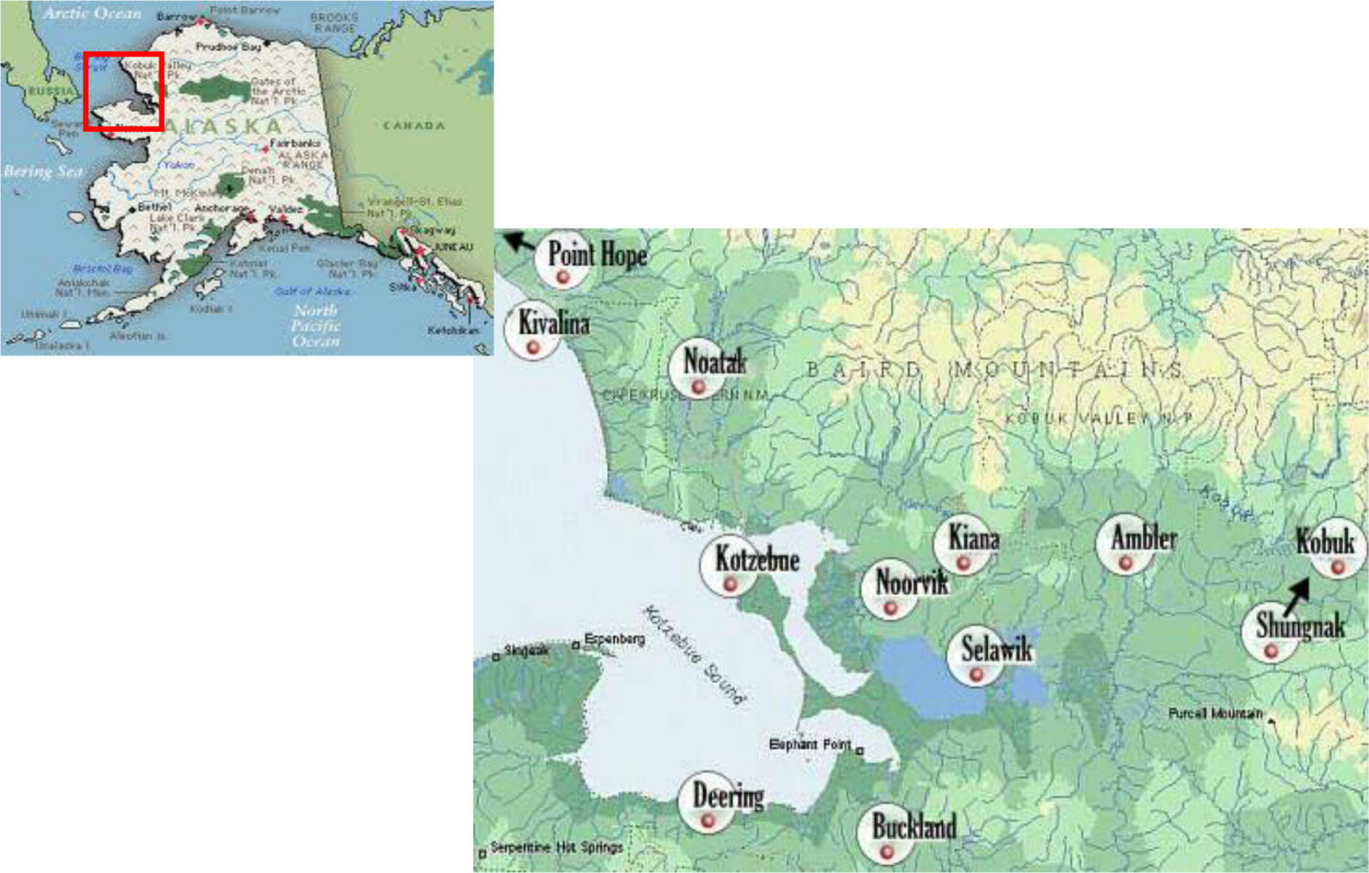
Maniilaq Service Area. Source: Alaska Map: www.loneyplanet.com; Maniilaq Service Area Map: www.maniilaq.org/aboutNWAlaska.html.

As “… collecting data by survey or primarily structured interviews is fundamentally inadequate” (21), p. 226) participant observation, combined with standard structured means of data collection is used to create a holistic representation of the processes involved in birth and maternal care among the participants in the study area (Fig. 1).

### Study area geography

The study population resides within the Maniilaq Service Area (MSA), located in Northwest Alaska (Fig. 1). No roads connect it with the rest of Alaska, and no roadways connect any of the villages with each other, so transportation can be an issue for the MSA, especially for health care. The Ralph Wien Memorial Airport in Kotzebue supports daily jet service to Anchorage and Nome, as well as smaller prop-driven aircraft to the villages. High winds can also shut down air travel and make any transport – especially for trips to deliver in Kotzebue or Anchorage – difficult.

The MSA experiences temperatures from about −10° F in February to highs in July of about 60° F, with rare low and high extremes of −82 to 86° F. Snowfall averages about 47 inches per year. From 2 June 2 to 9 July every year, the sun does not set, with the area's hours of daylight dwindling to almost full darkness for the winter.

### Population

Iñupiat mothers (aged 18 and older) residing in the selected MSA villages that gave birth at a regional hub or urban hospital in Anchorage, with their families and communities included in the study. Strategic sampling was used to identify mothers willing to participate.

### Study period/scope

Scoping and planning were initiated with Kotzebue and Buckland (2007–2009), and the project expanded to include Point Hope in 2009.

### Study villages

*Buckland* was selected as a comparison study village primarily because contacts lived here. The area also ended up serving as a perfect example of different expressions of western and Iñupiat values; and population (429 in 2011). This site houses a village clinic and has just recently started making provisions for access to running water beyond the washeteria (community laundry facility) and other public buildings (including the school).

*Kotzebue* is home to Maniilaq Health Center and former location of a now-closed Pre-Maternal facility. Its location and larger population (slightly over 3,000 in 2011) made for easier access – both in terms of travel logistics and (originally thought) garnering participation.

*Point Hope* (population ca. 680 in 2011) was also selected based on access to willing participants, and its unique identity as a coastal whaling community of NW Alaska. It is the only MSA village not under the Northwest Arctic Borough jurisdiction, and a Community Health Aide from the North Slope Borough (to which Point Hope belongs) still serves here.

### Analysis

Content analysis (22) was conducted to search for common themes in an inductive, recursive fashion. Using the themes liminality and communitas (6,7) and exploration of the presence of embodiment (10) of health in communities, this study links expressions of Iñupiat values to paths towards community-accepted maternal health care practices of each study village community. Prevalent themes expressed describing the birthing situation for each mother were noted, along with the level of adherence and acceptance of perceived risk assessment (and resulting transport determination). These components were then cross-checked with the age, era and manifestation of Iñupiat values of her home village to look for possible linkages.

The community participatory research portion of the study acquired UAF Institutional Review Board and participating Alaska Native Village Council approvals and appropriate informed consent (in compliance with *Principles for the Conduct of Research in the Arctic* (23)).

## Results

### Births

Latest available figures indicate that 239 births to Alaska Native mothers from different locations throughout the MSA occurred in 2009 (24). Nearly 90% of these births involved the mothers leaving their village.

Only 21 of 77 (28%) Alaska Native Kotzebue mothers gave birth in Kotzebue for a “local” birth, and 54 Alaska Native Kotzebue mothers and 101 other Alaska Native Maniilaq region mothers (from 11 remaining Maniilaq region villages), delivered live babies in Anchorage. Birth records (24) show deliveries at Kotzebue by mothers from other MSA villages rapidly trending downward since the height of facility usage in 1990, so that is the cut-off year for the Early era (or second generation) deliveries shown in Table I.

**Table I:** Study population by ANVMT policy (birth) era and village

	Village			
		
Birth era^a^	Buckland	Kotzebue	Point Hope	Total
Pre-policy (<1983)	2	1	2	5
Early (1984–1990)	2	2	3	7
Recent (1991–2011)	3	2	4	9
Total	7	5	9	21

a Defined by year mother experienced first birth.

### Eras (generations)

Twenty-one primary study participants from the MSA included 7 mothers (aged 19–68) from the Village of Buckland; 5 mothers (aged 22–64) from the Village of Kotzebue; and 9 mothers from the village of Point Hope (aged 19–75).

### Embodiment and Iñupiat values

With the biomedical view of birth becoming the norm here, many reported that access to these interventions, in general, is expected and welcome. One Buckland Elder (over 60 years old), herself a former traditional midwife, expressed a feeling that the “machines and medicines” available in Anchorage were for the best for the generations born during policy eras.

The stronger biomedical view of birth here also seems to be connected to the personal risks involved with the lifestyles of some of the younger mothers (smoking, drinking), combined with a greater sense of perceived risk associated with birthing outside of an urban hospital. Most of the birth and transport experiences relayed by Buckland mothers seemed very accepting of their need to transport, despite coming from a community featuring somewhat open expression of Iñupiat values Table II).

**Table II:** Present features of Iñupiat value system in MSA study villages

Village	Language Usage throughout village	Cultural Participation	Alaska-Native based village activities
Buckland	Signs in schools and buildings in Iñupiaq Used intermittently by village officials Songs dances in schools reflect youth knowledge of language Iñupiaq used intermittently by youth and Elders in everyday language	Very evident importance of Iñupiat-based celebrations from dancing and feasts to funerals Funerals, while western-religious based, still carry on traditions of Iñupiat meaning No mention, however, of blanket tosses Naming of babies still include pre-colonial naming practices and role expectations Uncles are still very much a part of a young man's hunting and fishing tutelage	Basketball seems to loom large in terms of carrying on a community-wide healthy competition and identity Fish camps are very important here Many subsistence-based families An active village council with older members. Some evidence of younger community member involvement in continuation of Iñupiat value system.
*Kotzebue*	Signs in schools, Maniilaq Health Center and office buildings appear in English and Iñupiaq Colloquial Iñupiaq-based terms heard in casual conversation, especially among Elders Local crafts sold in village office Church services in Iñupiaq and English Gatherings of Elders tend to “break out” in Iñupiaq; some grandparents teaching grandchildren Iñupiaq materials available at library on request Iñupiaq course taught as on-site and distance education through UAF Chuckchi Campus in Kotzebue	Parades with Iñupiat themes Some ceremonies with Iñupiaq terms Subsistence hunting fishing evident in village While clothing worn by adolescents appear more Abercrombie and Fitch than pre-colonial, there are still kuspuks and whaling parkas worn by older community members and infants and children are still occasionally dressed in the pre-colonial garments Participation in fish camps, fishing, caribou and seal hunting Whaling is more of an outer-village activity Modern village hotels reflect culture (*umiak* covers entry way) Fishing and seal-hunting still actively practiced by many in the village with young people encouraged to participate Trips to outer-villages are deemed as important to trips to Anchorage or Fairbanks Recent National Park Service museum presents cultural relics, uncertain impact on Alaska Native participation	Gathering/Iñupiat Community-building: Loss of important gathering place (senior centre in late 2010) that had invited informal congregation of youth and Elders Changing open hours of MHC Bingo and radio station are gathering places for select groups Occasional formal gatherings in churches around Western Christian religious–based holidays sometimes take on distinctly Iñupiaq features Iñupiat *Ilitqusiat* (statement of values) posted in village office, schools, and in social service areas Airports (1 major airline airport, 2 bush plane facilities) have evidence of Iñupiat-based community. Village stores (A/C in particular) are common meeting places for village community members Take-out food from increasing number of restaurants (and use of taxi service instead of walking) Shuttle bus service provided for Elders and those associated with Maniilaq
Point Hope	Village leaders and most community members of all ages possess working knowledge (speaking, reading and writing) of Iñupiaq language Not as many public buildings in this small village, so not as many signs outright noticeable Tikigiq school (PK-12), part of the North Slope Borough School District has signs in and English and NS school district has Iñupiaq language lessons and word search on their website	Village leaders take on more of an active role in the daily lives of the community residents Frequent ceremonies Continue to practice whaling ceremonies, rites and rituals passed down from generation to generation Whaling captain burial evidenced by landmark whalebones protruding from gravesites Remains of whalebone-constructed houses (which were lived in in this lifetime) still evident near shore Active carving, collection and use of pre-colonial, and ceremonial gear Strong affiliation exhibited between Christianity and Alaska Native spiritualism in this community Young people exhibit reverence for and are mindful of cultural taboos and expectations	Community activities continue to revolve around subsistence practices Everyday meals tend to include more subsistence food Social activities and caring responsibilities continue to adhere to Iñupiat value system-based protocols Iñupiat value system protocols still followed, even with presence of western-based conveniences and institutional structures (village clinic, fire dept., etc.) Village leaders and elders more directly involved in daily lives of community members, young and old.

*Source:* Compiled by author for use in study analysis (derived from ref. 15, p. 395).

The manifestation of cultural values (and related liminality, communitas and embodiment) associated with each village seemed to influence mothers’ and families’ prevailing worldviews concerning perceived risks and capacity for women to birth in their own community.

Kotzebue, the regional hub of the area, figures as openly expressing western values among the study villages. Buckland remains in many ways a community, openly expressing Iñupiat values. On the other hand, this remote village has social, organizational and political ties to Kotzebue that reflect western values that make it different from Point Hope, a whaling community where Iñupiat cultural dictates prevail in everyday life.

One second-generation Point Hope mother, in fact, relayed a birth story that involved her sending her brother for help as she was in labour with her second birth at home. On his return with her local birth attendant, he was greeted with her new-born baby's cry, she said, adding with a laugh, “I just leaned against the sofa and said [to herself] ‘I guess I'm gonna do this like they did a long time ago’.”

The living situations (working, married and unmarried, living with partner, or family) of the participants varied evenly across villages and age groups. Some younger groups of Point Hope mothers expressed frustration at having to be alone at birth: “They [hospital personnel] wouldn't let my husband stay in the room with me even though he had to be at the hospital, too.” If mothers do go to Anchorage (as required by risk assessment), and then get called back home for an emergency before delivery, the return flight is not paid for.

A young recent policy-era Point Hope mom, expecting her second child and planning on staying in her community to give birth while being monitored by a community health aide, opted not to discuss early pre-transport based on her assumption that all her “vitals” (blood pressure, blood sugar, baby's heart rate, etc.) were fine. As she went into early labour and reported this to clinic personnel, it was determined that she was experiencing preeclampsia, and she was immediately medevac'd to Anchorage. Once at Alaska Native Medical Center (ANMC), right after she successfully delivered her baby, she reported, a nurse chastised her for not coming to Anchorage earlier, even threatening this participant with a lawsuit for not submitting to earlier transport (5): “I didn't know I had preeclampsia. I was never told. If they [community health aide] had told me, I would have gone … but it [this experience] was really bad when that nurse started yelling at me right after my baby was born.”

## Discussion

This study reveals an important interplay between manifestations of Iñupiat values and desire and lengths gone to by indigenous women of NW Alaska (from different eras and locations) to participate in a more biomedical mode of birth. Women who adhere more closely to the model set up through the organized health care system operating in their area, seem to have more favourable experiences navigating the system than those who attempt to input their own views into the discussion of how things should go (1). Patients from outer-lying villages, with typically deeper levels of self-determination, can be viewed as difficult by medical staff at either the Maniilaq Health Center in Kotzebue or the ANMC in Anchorage. Many participants from each village, Buckland mothers in particular, refer to “nice nurses” at ANMC and “good food” at the Anchorage Pre-maternal home.

The problem for some mothers coming to Anchorage can involve typical “out-of-towner” issues like navigating the bus lines, boredom and other non-maternity care issues. Problems for Point Hope mothers of Early and Present policy eras, seem to lie more in what information is (or is not) passed on to patient–clients, for them to make their own decisions. Also, outside parameters (other children left at home, arrangements once the baby is born) vary among different locations and mothers. Women from Point Hope tend to have stronger ties with the North Slope area. However, because of quicker air transport to Kotzebue and Anchorage, Point Hope is MSA's only non-Northwest Alaska Native Corporation village. Frustration over poor communication experienced by mostly second and third generation Point Hope women could also be a result of these social and political divides.

### Maternal and native identity

Birthing women everywhere tend to be subject to surveillance, induction and caesarean deliveries are becoming more commonplace (25,26). Any objections to procedures tend to be met with anything from scare tactics to blame placed for child endangerment (27–31). Add to that the stereotypes of indigenous mothers (32–34), in general, and Alaska Natives (35,36), in particular, and the stress of motherhood in this area can become even greater.

### Expression of desired access to transport

Where features of Iñupiat values are openly present, a community-oriented birthing (and perhaps, health care) will be more likely sought, lessening the reliance on the existing transport policy. Conversely, where expression of western values are openly present in the community; the less likely alternative maternity care measures will be sought, and the greater risk will be leniently assessed, with further reliance on a bolstered transport system.

### Implications

Point Hope's population exhibit the characteristics of a village in the liminal stage most likely on the verge of transition stages toward self-determination – a community featuring strong expression of Iñupiat values with perhaps, more motivation to seek change. Like their Canadian Inuit counterparts of Nunavik, movements toward self-determination seem driven by a strong sense of community of a self-determined nature.

This study highlighted the call of Point Hope mothers for more communication and greater input into the decision-making process. Mothers from MSA study villages (Buckland) with more ties to larger bureaucracies, and western value systems (Kotzebue) are more likely to acquiesce to policy, and “make the best” of their prenatal travel. The dropping utilization of Kotzebue's Maniilaq Health Center for births, however, indicate a need for more informed dialogue with these apparently satisfied, but possibly over-treated birthing clients.

## Conclusions

This article addressed the lack of qualitative information concerning the maternal care services as utilized by the NW Alaska Native Iñupiat population. Ethnographic information regarding MSA mothers’ navigation of transport system during 3 birth eras was gathered and compared with descriptive features of their representative villages' expressions of Iñupiat values. A linkage between presence of Iñupiat values and sense of embodiment was discovered, along with differences in the ways these Buckland, Kotzebue and Point Hope mothers navigate the maternal transport system.

## Footnotes

1 The term *Iñupiat* refers to a group of north and northwest-dwelling Alaska Native Inuit peoples. The term *Iñupiaq* refers to their language or is used as a descriptive adjective meaning “of the Iñupiat.”
